# BreathDx – molecular analysis of exhaled breath as a diagnostic test for ventilator–associated pneumonia: protocol for a European multicentre observational study

**DOI:** 10.1186/s12890-016-0353-7

**Published:** 2017-01-03

**Authors:** Pouline M. P. van Oort, Tamara Nijsen, Hans Weda, Hugo Knobel, Paul Dark, Timothy Felton, Nicholas J. W. Rattray, Oluwasola Lawal, Waqar Ahmed, Craig Portsmouth, Peter J. Sterk, Marcus J. Schultz, Tetyana Zakharkina, Antonio Artigas, Pedro Povoa, Ignacio Martin-Loeches, Stephen J. Fowler, Lieuwe D. J. Bos, on behalf of the BreathDx Consortium, Waqar Ahmed, Antonio Artigas, Lieuwe D. J. Bos, Marta Camprubi, Luis Coelho, Paul Dark, Alan Davie, Emili Diaz, Gemma Goma, Timothy Felton, Stephen J. Fowler, Royston Goodacre, Hugo Knobel, Oluwasola Lawal, Jan-Hendrik Leopold, Ignacio Martin-Loeches, Tamara Nijsen, Pouline M. P. van Oort, Pedro Povoa, Craig Portsmouth, Nicholas J. W. Rattray, Guus Rijnders, Marcus J. Schultz, Ruud Steenwelle, Peter J. Sterk, Jordi Valles, Fred Verhoeckx, Anton Vink, Hans Weda, Tineke Winters, Tetyana Zakharkina

**Affiliations:** 1 Institute of Inflammation and Repair, University of Manchester, Oxford Road, Manchester, M13 9PL UK; 2 Philips Research, Eindhoven, The Netherlands; 3 Salford Royal NHS Foundation Trust, Greater Manchester, UK; 4 University Hospital of South Manchester NHS Foundation Trust, Manchester, UK; 5 Manchester Institute of Biotechnology (MIB), School of Chemistry, University of Manchester, Manchester, UK; 6 Intensive Care, Academic Medical Centre, University of Amsterdam, Amsterdam, The Netherlands; 7 Critical Care Department, CIBER Enfermedades Respiratorias, Corporacion Sanitaria Universitaria Parc Tauli, Sabadell, Spain; 8 Hospital de São Fransisco Xavier, Centro Hospitalar Lisboa Ocidental, Lisbon, Portugal; 9 Department of Clinical Medicine, St James’s Hospital, Multidisciplinary Intensive Care Research Organization (MICRO), Trinity Centre for Health Sciences, Dublin, Ireland

**Keywords:** Ventilator-associated pneumonia, Breath analysis, Volatile organic compounds, Metabolomics, Sensitivity, Specificity

## Abstract

**Background:**

The diagnosis of ventilator-associated pneumonia (VAP) remains time-consuming and costly, the clinical tools lack specificity and a bedside test to exclude infection in suspected patients is unavailable. Breath contains hundreds to thousands of volatile organic compounds (VOCs) that result from host and microbial metabolism as well as the environment. The present study aims to use breath VOC analysis to develop a model that can discriminate between patients who have positive cultures and who have negative cultures with a high sensitivity.

**Methods/design:**

The Molecular Analysis of Exhaled Breath as Diagnostic Test for Ventilator-Associated Pneumonia (BreathDx) study is a multicentre observational study. Breath and bronchial lavage samples will be collected from 100 and 53 intubated and ventilated patients suspected of VAP. Breath will be analysed using Thermal Desorption – Gas Chromatography – Mass Spectrometry (TD-GC-MS). The primary endpoint is the accuracy of cross-validated prediction for positive respiratory cultures in patients that are suspected of VAP, with a sensitivity of at least 99% (high negative predictive value).

**Discussion:**

To our knowledge, BreathDx is the first study powered to investigate whether molecular analysis of breath can be used to classify suspected VAP patients with and without positive microbiological cultures with 99% sensitivity.

**Trial registration:**

UKCRN ID number 19086, registered May 2015; as well as registration at www.trialregister.nl under the acronym ‘BreathDx’ with trial ID number NTR 6114 (retrospectively registered on 28 October 2016).

## Background

Ventilator-associated pneumonia (VAP) is a frequent complication of mechanical ventilation in the Intensive Care Unit (ICU) [1–3] and the associated morbidity results in substantial healthcare costs [4, 5]. The diagnosis of VAP remains challenging as clinical, laboratory and radiological parameters are sensitive but non-specific for VAP and suffer from high inter-rater variability [6, 7]. A lower respiratory tract sample [bronchoalveolar lavage (BAL), endotracheal aspirate or protected specimen brush sample] is recommended for microbiological confirmation of clinically suspected VAP [8], but these results take days to become available and the procedures cannot be repeated frequently due to their invasiveness. As a result of this delay, patients are overtreated with antibiotics, as empiric antibiotic treatment is initiated immediately after obtaining a lower respiratory tract sample. Subsequent microbiological results help to tailor and deescalate antibiotic treatment [9], so the lower respiratory tract sample continues to be of crucial importance for diagnosing VAP.

There is need for a less invasive and more time-efficient diagnostic technique that ultimately reduces the amount of antibiotics used to treat suspected VAP. Clinical scoring systems, like the Clinical Pulmonary Infection Score (CPIS) [10] and biomarkers have been studied as means to exclude VAP, but so far these attempts have not resulted in a test that is suitable for current ICU practice [11–15].

Exhaled breath contains volatile organic compounds (VOCs); small volatile molecules that result from host or bacterial metabolism or are contaminants from the environment [16, 17]. Exhaled VOC profiles have been shown to differentiate between many different disease states and may therefore qualify as non-invasive biomarkers [18–21]. Capture of VOCs and exhaled breath analysis has proven to be safe and reliable in mechanically ventilated critically ill patients [22–24]. Data from in-vitro experiments suggest that the presence of bacteria may be detected based on a small panel of VOCs [17]. This concept was recently translated in vivo: ventilated patients with and without positive bacterial cultures of endotracheal aspirate could be discriminated based on exhaled VOCs [24].

The aim of this study is to determine whether molecular analysis of breath can be used to discriminate between patients that are suspected of VAP who have positive cultures and who have negative cultures with high sensitivity, thus having the potential to limit antibiotic use. Secondly, we hypothesize that molecular analysis of breath can be used to specifically detect the causative pathogen in patients that are suspected of VAP, offering the possibility of more targeted antibiotic therapy.

## Methods

### Design

‘BreathDx – Molecular Analysis of Exhaled Breath as Diagnostic Test for Ventilator–Associated Pneumonia’ is an international European multicentre observational cohort study in intubated and ventilated ICU patients suspected of VAP. Six ICUs of university hospitals in the Netherlands, the United Kingdom, Spain and Portugal are involved: the Academic Medical Centre (AMC) in Amsterdam; University Hospital South Manchester (UHSM), Salford Royal and Central Manchester University Hospitals in Manchester; Parc Tauli Hospital in Sabadell; and Sao Francisco Hospital/Nova Medical School in Lisbon. Patients are expected to be recruited from all six sites over an 18 to 24-month time period. The project is funded by the European Union (*BreathDx – 611951*).

### Study population

Patients at one of the six involved ICUs that are clinically suspected of having VAP are eligible for the study. VAP is defined by (1) systemic changes [temperature >38 or <36.5 °C; white blood cell count <4,000 or >12,000/mm^3^]; and (2) chest abnormalities [new infiltrates on chest X-ray, purulent tracheal secretions]; and (3) positive microbiology results [25]. Inclusion criteria are (1) 18 years and older and (2) intubation and mechanical ventilation for > 48 h and (3) clinical suspicion of VAP (aforementioned systemic changes combined with chest abnormalities). Exclusion criteria include patients who: (1) are deemed clinically inappropriate to collect samples from (e.g. if they are receiving end-of-life care); or (2) are in strict isolation (e.g. Middle East Respiratory Syndrome, Ebola or resistant tuberculosis).

### Study procedures

Patients will be recruited and samples collected within 24 h of the clinical suspicion of VAP. First breath samples will be collected, followed by bronchoscopy and bronchial lavage. Standard Operating Procedures (SOPs) will be in place at all sites in order to ensure samples are collected equally. Breath samples will be shipped within days after collection and shall be analysed within 2 weeks upon arrival. Previous results have shown breath samples can be stored for at least 14 days without loss of data [26]. The (mini) BAL samples are processed and frozen immediately after recruitment. When all (mini) BAL samples are collected they will be shipped on dry ice to remain conserved.

### Breath sampling

**Fig. 1:**
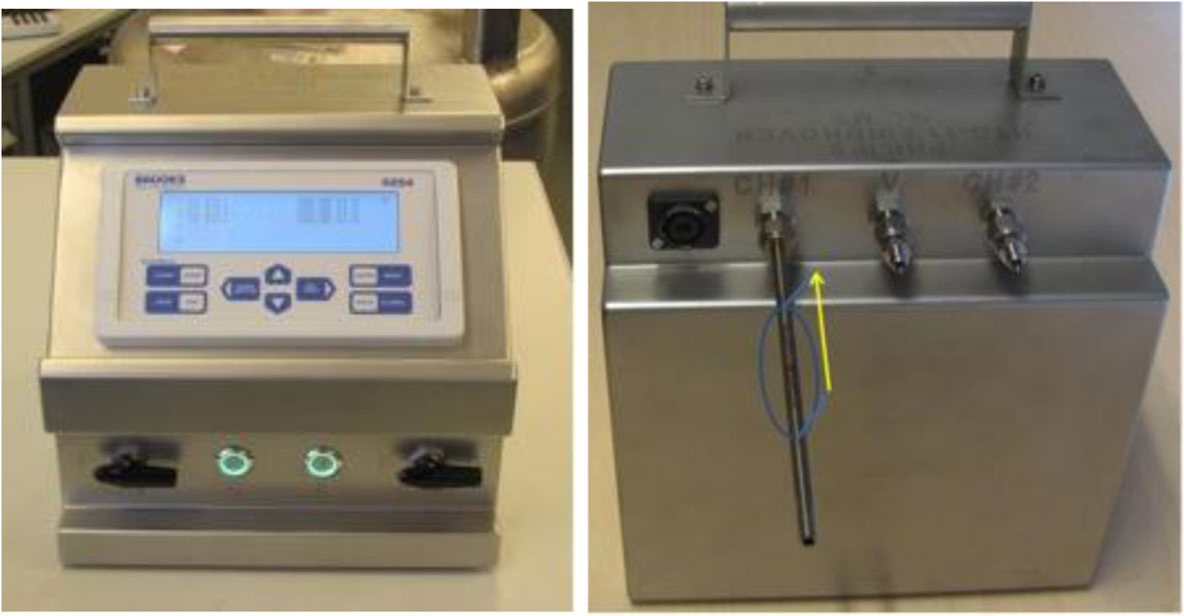
The breath gas sampler

Breath samples will be collected once (at time of recruitment) using a breath gas sampler (BGS, see Fig. 1) consisting of a pump (NMS020B 6VDC Micro Membranegas pump), a mass flow controller (Horiba STEC Z500), battery and charger (Panasonic LC-RA1212PG and IDEAL POWER PC170-2) all combined in a metal casing with operating display (Brooks Instrument 0254). Using this BGS and PTFE (PolyTetraFluoroEthylene) tubing (Swagelok, Warrington, UK), the exhaled breath is drawn from the sidearm of a T-piece connector inserted in the ventilator circuit distal of the HME filter and through a stainless steel sorbent tube (Markes International, Llantrisant, UK; and Gerstel, Mülheim an der Ruhr, Germany), adapted from Bos et al [23]. Subsequently the sorbent tubes will be transported for off-site analysis. The samples will be link-anonymised. Two pairs will be collected per patient and will be sent to two different laboratory locations for analysis (one pair to Philips Research, Eindhoven, the Netherlands and the other to Manchester Institute of Biotechnology, University of Manchester, Manchester, United Kingdom). For analysis at Philips Research the exhaled breath is collected using sorbent tubes packed with 300 mg Carbograph 5TD (Markes International, Llantrisant, UK) and 90 mg Tenax GR (Sigma-Aldrich Chemie B.V., Zwijndrecht, the Netherlands). The samples to be analysed at the Manchester Institute of Biotechnology are collected using sorbent tubes packed with 200 mg Tenax GR (Markes International, Llantrisant, UK). All samples are taken in duplicate. Breath samples are stored in a cold room immediately after collection. This sampling setup has shown to be safe and adequate for sample collection in ventilated ICU patients [18, 23, 27].

### Bronchoalveolar lavage

**Fig. 2:**
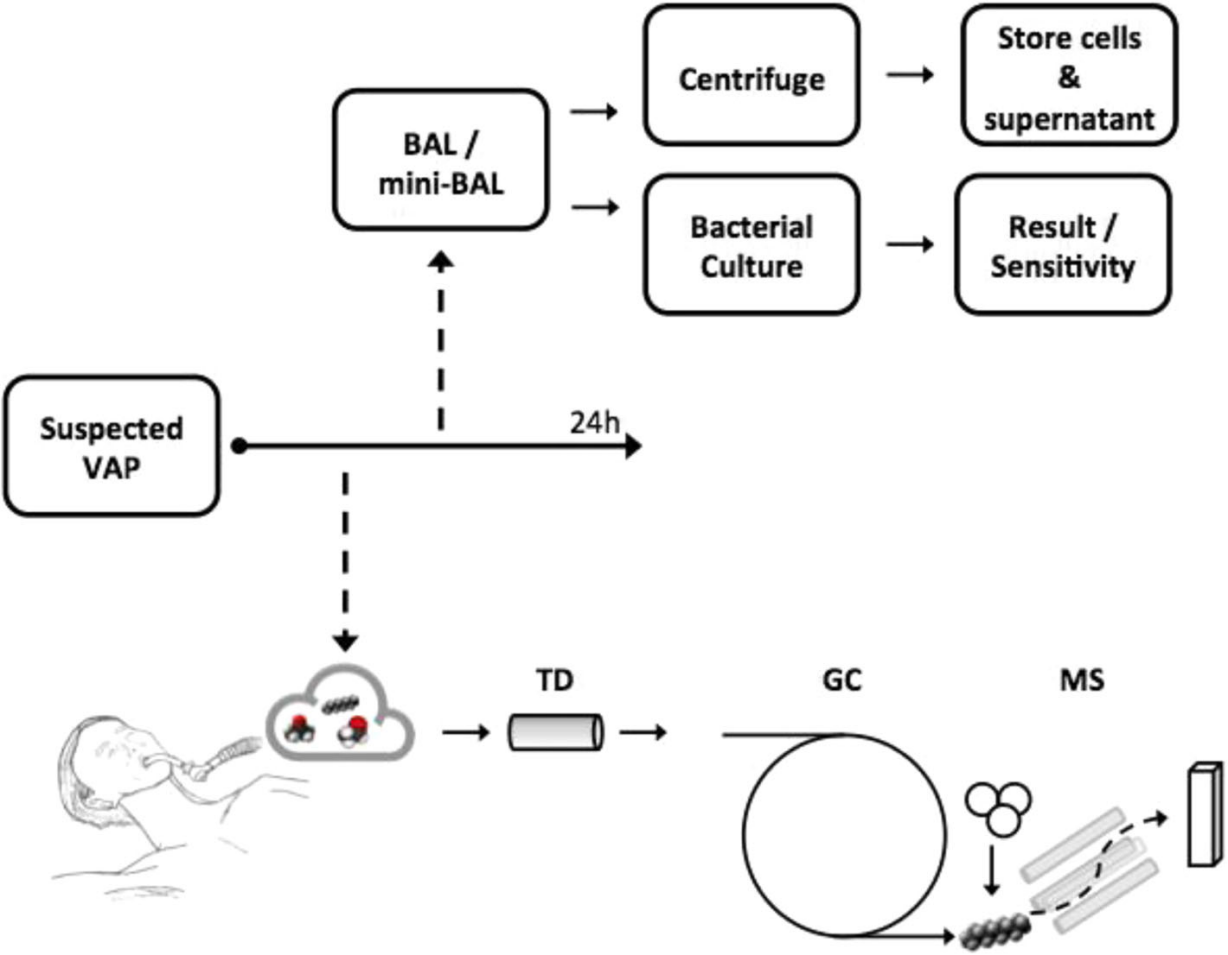
Overview of the sample collection

A (mini)-BAL sample will be obtained for microbiological analysis as soon as possible after collecting the breath samples (see Fig. 2). A syringe is connected to a bronchoscope or a 50 cm suctioning catheter and 20 mL 0.9% saline is injected in the airway. At least 4 mL is aspirated of which 1 mL is sent to the medical microbiology for routine cultures, leading to a semi-quantitative bacterial count with a cut-off of 10^4^ colony forming units/mL defining a positive culture. An aliquot of the (mini)-BAL sample will be processed and stored for future analysis.

### Gas chromatography and mass-spectrometry

The exhaled breath sample will be analysed using Thermal Desorption – Gas Chromatography – Mass-Spectrometry (TD-GC-MS). In order to separate, quantify and identify a wide range of volatiles in breath, different chromatographic set-ups at Eindhoven and Manchester are used. Both GC-MS analyses will result in a list of detected volatiles and their relative concentrations.

At Philips Research, the sorbent tubes are thermally desorbed at 225 °C (TDSA, Gerstel, Mülheim an der Ruhr, Germany) into the GC capillary column. Solvent venting mode is used to transfer the sample without loss to the packed liner (filled with Tenax TA) held at −55 °C which is subsequently heated to 280 °C. A cold trap (CTS2, Gerstel, Mülheim an der Ruhr, Germany) is used to minimize band broadening (initial temperature −150 °C, after 1.6 min heated to 220 °C). A capillary gas chromatograph (6890 N GC, Agilent, SantaClara, CA, USA) using a VF1-MS column (length 30 m × internal diameter 0.25 mm, film thickness 1 μm, 100% dimethyl-polysiloxane, Varian Chrompack, Middelburg, the Netherlands) is used with the following temperature program: 30 °C-hold 3.5 min, ramp 5 °C/min to 50 °C, hold 0 min, ramp 10 °C/min to 90 °C, ramp 15 °C/min to 130 °C, ramp 30 °C/min to 180 °C, ramp 40 °C/min to 280 °C, hold 1 min. A Time-of-Flight Mass Spectrometer (Pegasus 4D system, LECO, St. Joseph, Mi, USA) is used in the electron ionization mode at 70 eV, with a scan range of m/z 29–400 Da, scanning rate 20 scans/s. Gaseous calibration standards (10 ppmv acetone-D8, hexane-D14, toluene-D8, xylene-D10 in nitrogen, Air Products, Amsterdam, the Netherlands) are made by use of a home-built dilution system and loaded on adsorption tubes as an internal standard.

At Manchester Institute of Biotechnology sorbent tubes filled with Tenax GR are thermally desorbed at 280 °C (TD100, Markes International, Llantrisant, UK) into a cold trap to minimize band broadening (initial temperature −0 °C, after 2 min heated again to 280 °C). This will then be fed into a capillary gas chromatograph (7890B GC, Agilent, SantaClara, CA, USA) using a HP-5 ms Ultra Inert column (length 30 m × internal diameter 0.25 mm, film thickness 0.25 μm, (5%-Phenyl)-methylpolysiloxane, Agilent, SantaClara, CA, USA) with the following temperature program: 40 °C - hold 0 min, ramp 6 °C/min to 170 °C, hold 0 min, ramp 15 °C/min to 190 °C for a total time of 23 min. A Triple-Quad mass spectrometer (7010, Agilent, SantaClara, CA, USA) will be used in the electron ionization mode at 70 eV, with a scan range of m/z 40–500 Da, scanning rate 4 scans/s. A gaseous calibration standard (1 ppmv, 4-Bromofluorobenzene in nitrogen, Thames Restek, UK) will be loaded on adsorption tubes as an internal standard for 1 min at 20 ml/min. Additionally, to aid in retention time correction, an external standard containing a mixture of laboratory standard VOC chemicals (Sigma Aldrich, UK) will be sampled on separate tubes, either side of a breath sample.

### Clinical data

Clinical data regarding patient characteristics, primary and secondary diagnoses, comorbidities, drug history, measures of disease severity such as Acute Physiology and Chronic Health Evaluation (APACHE) IV [28] and Simplified Acute Physiology Score (SAPS) II [29] ventilation data, CPIS [10], culture data, outcome variables (ICU/hospital length of stay, mortality) and adverse events will be collected.

### Study outcomes

The primary endpoint is the accuracy of cross-validated prediction for positive respiratory cultures in patients that are suspected of VAP, with a sensitivity of at least 99% (high negative predictive value).

The secondary endpoints are: (1) the accuracy of cross-validated prediction for growth of a specific pathogen with a specificity of at least 90%; (2) GC-MS identified molecular markers that can distinguish between patients with and without microbiologically confirmed VAP with *p* <0.05 and a false discovery rate <0.05; (3) GC-MS identified molecular markers that can distinguish between patients with and without growth of specific pathogens during bacterial culture with *p* <0.05 and a false discovery rate <0.05; (4) accuracy of prediction within the subgroup of patients with and without a previous respiratory infection; (5) accuracy of prediction within the subgroup of patients intubated for less and more than 1 week.

### Sample size calculation

The sample size calculation has been performed based on binomial distributions instead of normal approximations to this distribution [30]. With an expected sensitivity of 99% (almost 100% negative predictive value) we require the lower 95% confidence limit to be larger than 90% with 95% probability. 90% sensitivity is the absolute minimal in a discovery study such as this, for a lower sensitivity is clinically irrelevant and would not be clinically useful. With these figures, the required number of cases is estimated to be 61 [30]. Assuming a prevalence of 40% of positive cultures of bronchoalveolar lavage in patients that are clinically suspected of VAP [24], the total study sample size should be 153 subjects.

### Statistical analysis

The GC-MS data are three-dimensional in nature, with ion counts for every combination of m/z value and retention time. The chromatogram represents the total ion count (TIC) measured by GC-MS as a function of retention time. The first step in pre-processing consists of correcting the chromatographic baseline, required for proper estimation of ion intensities and accurate molecule identification based on the mass spectra. Subsequently the data will be visually inspected to exclude contaminated samples. Contamination of the sampling tubes (e.g. due to loose fittings during transport) can severely distort the stored breath content. These tubes will be excluded from further analysis.

For peak detection we will use the method described by Smith et al. [31] implemented in the R-package XCMS [31–33]. It is currently the most cited pre-processing tool in the metabolomics literature [34]. The settings for peak detection will be determined as described by Smith et al. [31], using model peak widths that are considerably larger than the signal peak (1.5 – 4 times) for consistent signal-to-noise improvement [35].

The intensity of the internal standards toluene-d8 and 4-bromofluorobenzene will be used to normalize all other peaks in the GC-MS data of the two laboratories, respectively.

The retention time alignment in the XCMS package works very well for relatively small retention time shifts. For large shifts this method becomes inaccurate resulting in the loss of peaks in the final table and erroneous alignment of the samples. In our experiment the samples will be measured over a time span of at least 18 months. Therefore rather large retention time shifts can be expected. To account for the retention time shifts the following approach will be applied, which consists of two steps [27]. First the major part of the time shifts will be corrected by using anchor points (marker molecules), i.e. molecules with clearly identifiable mass spectra distributed over the full retention time window. Examples are isoprene, toluene and compounds from the internal standards. These molecules will be identified by comparing the measured mass spectra to the spectra published in the National Institute of Standards and Technology (NIST) chemistry web book database [36] using the dot-product function as similarity measure. According to Stein and Scott [37], this algorithm gives the best similarity estimate between mass spectra. The first raw retention time correction will be performed using a linear or quadratic fit to the retention times of the marker molecules. The second step in the approach will consist of fine alignment using the regular retention time correction of the XCMS package, as described by Smith et al. [31].

All the steps above will result in an ion-fragment peak table. Each row in the table corresponds with a sample. The first few columns will contain sample and patient data, such as sample data, age, gender and illnesses. The remaining columns will contain the abundances of the peaks or ion-fragments; typically there are a few thousand. This table will serve as input for extra quality checks and subsequent statistical analysis.

One of the quality checks will consist of comparing the two pairs of samples successively collected from each patient. The content of these duplicates should be equivalent, especially when compared to the content of other, arbitrary samples. Cosine similarity measures will be plotted into the histograms for duplicate samples and arbitrary samples. The equalities of the distributions in the histograms will be tested with the two-sided Kolmogorov–Smirnov test. Additionally the intensity of several common molecules between replicate samples will be analysed using Bland-Altman plots.

Samples measured on different GC-MS instruments are rarely identical due to multiple differing technical characteristics. Previous attempts to align data from different GC-MS machines have proved to be very complicated. Therefore the samples from different GC-MS machines will be aligned separately. For each GC-MS machine fragment averaging over the two consecutive samples will summarize peak intensities. In this way the number of breath features becomes roughly twice as large. Newly added features will be correlated to the existing features, since they are sequentially sampled from the same patient. The dependence between the features and the higher number of features puts higher strain on the statistical analysis.

The data can now be used for (1) data discovery, (2) untargeted analysis and (3) targeted analysis. Data discovery will consist of principle component analysis (PCA) on the log-transformed and scaled data, and Ward clustering on the 100 most abundant peaks eluting at least one second apart, as well as on the most relevant principle components.

Untargeted analysis will consist of building predictive models based on the data. The models will reduce the dimensionality of the dataset: the number of features is many times higher than the number of patients, increasing the risk of over-fitting. Additionally the features are not independent: several ion fragments originate from the same molecule. The statistical model needs to be able to deal with this. Finally breath data typically shows large variation in VOC abundance between, but also within individuals. Considering these characteristics of the data, we have chosen sparse partial least squares models to analyse the log-transformed data [38]. The small number of included patients will not allow data to be split into a training set and a validation set, although this is the preferred method. Instead permutation tests will be used to estimate the performance of the model.

In the targeted approach existing literature will be searched for potential biomarkers for VAP. The abundance of such molecules will be compared between patient groups using student t-tests or Mann–Whitney-Wilcoxon tests for normally and non-normally distributed data respectively. The important advantage of this approach is the low likelihood on false discoveries. The amount of comparisons is limited and previous findings will be validated.

In order to assess the influence of possible confounders (e.g. comorbidities, ventilator settings, medications) on the association between exhaled breath and the VAP the log odds ratios will be compared between a logistic regression model with the VOCs of interest as dependent variables and VAP (yes/no) as independent variable and the same model with the inclusion of the potential confounder as co-variate. When the log odds ratio shows a change of more than 10% the co-variate will be considered a confounder.

### End of study definition

The study will end when the required sample size is reached.

### Reporting

The results will be reported strictly following Standards for Reporting Diagnostic Accuracy (STARD) guidelines [39].

## Discussion

This manuscript describes the protocol for a multicentre prospective observational study that aims to develop a diagnostic tool for discriminating between patients that are suspected of VAP who have positive cultures and who have negative cultures through breath analysis using TD-GC-MS. Additionally, we aim to describe patterns of VOCs in exhaled breath that are predictive of the presence of specific pathogens. Ultimately we strive for a diagnostic test with 99% sensitivity for culture positive VAP, which is required in an ICU setting where delayed initiation of adequate antibiotic therapy is unacceptable.

Several clinical challenges can be recognized for this study a priori. First, the reported incidence of VAP has declined over the last decade [40]. As recruitment depends on the clinical suspicion of VAP, this could slow the inclusion rate. The clinical definition for inclusion into the study could also be seen as a weakness of the study as clinical practice may vary from hospital to hospital. However, we have tried to include hospitals from a wide variety of settings and countries throughout Europe to cover the heterogeneity in clinical practice. This geographical variation may also introduce noise into the data collected by breath analysis as the environment contributes to exhaled VOCs [41, 42]. Another challenge concerns the secondary aim of this study to identify patterns of VOCs that are predictive of the presence of specific causative pathogens. A number of VOCs are already associated with certain pathogens. There is a large number of pathogens that can cause VAP [43] and the groups of patients are not likely to be infected with the same pathogen. This is a risk that is inherent to a prospective clinical study. We expect to find sufficiently large groups of patients for at least the most important pathogens in VAP: *Pseudomonas aeruginosa*, *Staphylococcus aureus* and *Enterobacteriaceae* [43].

There are also multiple analytical challenges. Patients will be recruited over a minimum 18-month period. As a result, the GC-MS platforms will have to be stable over this period of time when there is potential for column degradation that can change the retention time of VOCs. Several members of the consortium previously performed studies over similarly long periods of time and have developed statistical tools to correct for this shift in retention time [27]. The sensitivity of the mass spectrometer may also drift. This was a problem in previous studies and therefore an internal standard was included in the present protocol. As in any study that focuses on breath analysis there is always the challenge of statistical overfitting [44]. We expect to find several hundreds of VOCs in the breath of patients. These will be used as predictors for the presence of VAP. Such a high dimensional predictor matrix easily results in false discovery and therefore sufficient internal validation measures must be taken [45]. There, we suggest two approaches in this protocol. First, we aim to validate previously found markers. This limits the number of statistical comparisons and increases the changes on valid discoveries. Second, novel biomarkers are discovered using cross-validation and permutation tests.

The described protocol also has several strengths and is pragmatic in nature. The studied population is clinically very relevant as a treatment decision may be influenced by the outcome of the test. Using an unbiased approach, with patient recruitment in multiple European countries and breath analysis on multiple GC-MS platforms, this study allows for the development of a test that is applicable in a wide variety of hospitals. Special attention was given to analytical versatility; multiple sorbent beds are used and breath is analysed on two separate platforms that have complementary analytical strengths. There is also additional intellectual benefit; the results may be translated to other patients; the identified markers may also be studied in patients suspected of community- or hospital-acquired pneumonia. Another possibility for value within the results is the development of a continuous breath test that can warn the clinician that a patient is about to develop pneumonia.

The results from this study will have direct clinical implications. If the sensitivity of 99% is reached while maintaining a moderate to good specificity, antibiotic treatment can be withheld from a large proportion of VAP-suspected patients. With a prevalence of culture positive VAP of 40%, 48 out of every 100 patients would benefit in the scenario that a sensitivity of 99% and a specificity of 80% is obtained. Antibiotic therapy could be withheld in four patients in such a case. These figures improve with increased specificity at the pre-selected sensitivity and a lower prevalence of culture positive VAP.

In conclusion, we hypothesize that breath analysis can be used for discrimination between VAP suspected patients with and without microbiological positive cultures with a high sensitivity, and can be used to specifically detect the causative strain of bacteria.

### Trial status

Patient recruitment for the BreathDx study is currently ongoing.

## Abbreviations

AMC: Academic Medical Centre
APACHE: Acute physiology and chronic health evaluation
BAL: Bronchoalveolar lavage
BGS: Breath gas sampler
CPIS: Clinical Pulmonary Infection Score
eV: electron volt
GC: Gas Chromatography
ICU: Intensive Care Unit
m/z: Mass-to-charge ratio
MS: Mass Spectrometry
NIST: National Institute of Standards and Technology
PCA: Principle component analysis
ppmv: Parts per million by volume
PTFE: PolyTetraFluoroEthylene
SAPS: Simplified Acute Physiology Score
STARD: Standards for reporting diagnostic accuracy
TD-GC-MS: Thermal Desorption – Gas Chromatography – Mass Spectrometry
TIC: Total ion count
UHSM: University Hospital South Manchester
UKCRN: United Kingdom Clinical Research Network
VAP: Ventilator-associated pneumonia
VOC: Volatile organic compound
